# Eczema Simplex Dependent upon Ametropia

**Published:** 1888-04

**Authors:** S. O. Richey

**Affiliations:** Washington, D. C.


### ECZEMA SIMPLEX DEPENDENT UPON AMETROPIA.

BY S. O. RICHEY, M. D.,
Washington, D. C.

In the Archives of Ophthalmology, Volume XIII, No. 1, 1884, may be found the report of a case of eczema of the malar prominences, whose exciting cause was an anisometropia, the eyes being hypermetropic to a different degree.

I have now to supplement that with a similar case, referred to me, from the same family, by my friend, Dr. C. E. Hagner.

October 8, 1886, Miss S. T., a cousin-german of C. K., consulted me, complaining of temporal pain, and pain at the nucha, almost continuous. Corrugated supercilia indicated the demand upon the circumorbital muscles for aid in ciliary adjustment. Frequently, the protracted use of the eyes upon objects at close range was followed by an eczematous eruption over the malar prominences. V. R. $= \frac{20}{xx}$: V. L. $= \frac{20}{xxx}$. Reads Sn. 1, at 12 inches.

Insufficiency of the external recti at 18 feet $=2°$. Eyes normal in other respects. Mydriasis reduced the vision of each eye to $\frac{20}{CC}$, which was restored to $\frac{20}{XX}$ by a +2. Ds each.

The lenses were worn constantly for some months, and then were laid aside for distance, though she still resorts to them for near vision. I have often seen the patient since to learn that the relief from the pain and eruption has been permanent.

These two cases only have occurred in my professional experience, and I can find no mention of any other. They are interesting for the aid they may afford in the study of the pathology of eczema.

